# Connecting the oxidation of soot to its redox cycling abilities

**DOI:** 10.1038/ncomms7812

**Published:** 2015-04-15

**Authors:** María Antiñolo, Megan D. Willis, Shouming Zhou, Jonathan P.D. Abbatt

**Affiliations:** 1 Department of Chemistry, University of Toronto, 80 St George Street, Toronto, Ontario, M5S 3H6 Canada; 2 Facultad de Ciencias y Tecnologías Químicas, Universidad de Castilla-La Mancha, Avda. Camilo José Cela s/n, Ciudad Real 13071, Spain

## Abstract

Although it is known that soot particles are emitted in large quantities to the atmosphere, our understanding of their environmental effects is limited by our knowledge of how their composition is subsequently altered through atmospheric processing. Here we present an on-line mass spectrometric study of the changing chemical composition of hydrocarbon soot particles as they are oxidized by gas-phase ozone, and we show that the surface-mediated loss rates of adsorbed polycyclic aromatic hydrocarbons in soot are directly connected to a significant increase in the particle redox cycling abilities. With redox cycling implicated as an oxidative stress mechanism that arises after inhalation of atmospheric particles, this work draws a quantitative connection between the detailed heterogeneous chemistry occurring on atmospheric particles and a potential toxic mechanism attributable to that aerosol.

## Abstract (web summary)

Although it is known that soot particles are emitted in large quantities to the atmosphere, our understanding of their environmental effects is limited. Here, the authors study hydrocarbon soot particles during oxidation by ozone, suggesting that this process leads to increases in redox activity.

## Graphical abstract

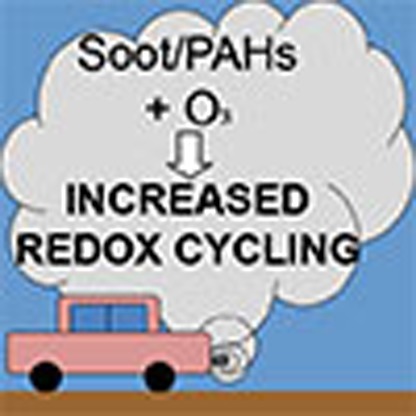

One of the main toxicological mechanisms resulting from atmospheric particulate matter (PM) inhalation is oxidative stress, arising from an imbalance of oxidants and antioxidants^1^,^2^. This imbalance can be produced by catalytic redox-cycling reactions, through species with labile oxidation states, which result in the depletion of reducing agents within the cell and the generation of reactive oxygen species (ROS). Likely catalysts that have been identified are transition metals and quinones, although other redox classes of compounds are also possible^3^,^4^,^5^. It is also shown that PM can lead to increased negative health effects after it has been exposed to atmospheric oxidants. For example, greater lung injury and inflammation were observed when rats were exposed to diesel exhaust particles (DEP) oxidized by O_3_ compared with non-oxidized DEP^6^. Other studies reported an increase of the response in redox cycling rates when different combustion particles were exposed to O_3_ (refs 7, 8, 9). Photoxidized DEP also caused greater inflammation and showed an increase in the redox activity^10^,^11^

Whereas these studies have identified important correlations, to move forward in this field it is necessary to develop a molecular-level understanding of the composition and atmospheric processing mechanisms of particles. As well, we must connect this detailed surface chemistry to the routes for PM toxicity, such as the ability of PM to promote redox cycling and ROS generation. To that end, the central hypothesis in this work is that reactive oxidation chemistry with soot particles involves interactions with either polycyclic aromatic hydrocarbons (PAHs), which are known to coat combustion particles, or with the chemically unsaturated graphitic soot backbone that has PAH-like character. In particular, it is well known that quinones are oxidation products of PAHs^12^,^13^,^14^,^15^, which are potential redox-cycling agents. Indeed, gas-surface oxidation of adsorbed anthracene by ozone has identified anthraquinone as a major oxidation product^16^.

The motivation to study soot particles is multifold. In addition to established correlations between black carbon and negative health outcomes^17^, from a chemical perspective quinones and hydroquinones have been detected on carbon black surfaces and in engine exhaust emissions^18^,^19^,^20^, and their high efficiency as electron transfer agents has been demonstrated^3^. As well, Shinyashiki *et al.*^21^ reported that most of the redox and electrophilic activity of the organic extracts of DEP is associated with quinone-like substances. Past studies have reported an increase in PM redox cycling activity when carbonaceous particles have been exposed to ozone^7^,^8^,^9^.

There are many kinetic studies addressing the reaction between soot and O_3_ where the primary focus has been on characterizing the overall loss rate of gaseous O_3_. In particular, the uptake coefficient, γ, describes the kinetics of this heterogeneous process, being the net probability that a molecule (O_3_ in this case) undergoing a gas-kinetic collision with a surface (soot in this case) is lost to the surface. γ values of ∼10^−3^ have been reported for freshly emitted soot and ∼10^−6^–10^−4^ for aged soot^22^. There are also kinetic studies for the exposure of various PAHs adsorbed on different surfaces to gaseous O_3_ (refs 13, 23, 24, 25). For example, the reported uptake coefficient for O_3_ with benzo[*a*]pyrene (BaP) adsorbed on soot was in the range 10^−6^–10^−5^ for O_3_ concentrations between 5.0 × 10^11^ and 2.5 × 10^13^ molecule cm^−3^ (ref. 23). Notably, the oxidation kinetics of PAHs that are formed as an intrinsic component of soot aerosol have not been studied.

In this work, we determine a relationship between the oxidation-driven chemical change of hydrocarbon soot particles and their redox cycling activity, demonstrating a quantitative connection of the atmospheric oxidative processing kinetics and mechanism to potential toxic effects of PM. Using a new aerosol mass spectrometric technique, we monitor the real-time loss of soot-bound PAHs as they are oxidized within an environmental chamber and determine the O_3_ uptake coefficient from the observed chemistry. We also make measurements of the redox activity of the increasingly oxidized soot particles^26^. In addition, we evaluate the redox cycling activity that arises when soot particles that are partially coated with benzo[*e*]pyrene (BeP), a PAH detected in ambient particles^27^, are exposed to O_3_. The aim of these latter experiments is to compare the surface chemistry observed for a single PAH species with the behaviour of a collective ensemble of PAHs present within combustion soot.

## Results

### Size characterization of soot particles

The size distribution of soot particles inside the chamber was measured with a scanning mobility particle sizer (SMPS) between 14.6 and 710.5 nm during the experiments for both bare soot and soot coated with BeP. No significant change in the size distribution was observed after BeP coating, consistent with the coating being sub-monolayer in average thickness. In Supplementary Table 1, average values for the mean mobility diameter, particle number concentration and total mass loading inside the chamber before starting the oxidation are presented with the mixing ratios of gas-phase species. The observed high mean diameter (>300 nm) arises from coagulation of smaller soot particles occurring during the time required to fill the chamber with the required mass loading to run the DTT assay (around 1 h 30 min). The soot particle effective density was also calculated from mobility and aerodynamic measurements and is shown in Supplementary Table 1^28^. When comparing mobility and aerodynamic diameters of the soot particles, it was found that the mobility diameter was always much higher than the aerodynamic diameter, indicating that the soot particles have a low effective density.

During an experimental run, the particles became on average 11% larger in mobility diameter, and their number concentration decreased on average 45%, with both effects likely arising from preferential loss or coagulation of small particles. Oxidation of the particles is unlikely to alter their size significantly, given that the oxidation occurs only at the surface, unless there is some morphological change occurring as well.

### Oxidation-driven compositional changes of soot particles

**Figure 1:**
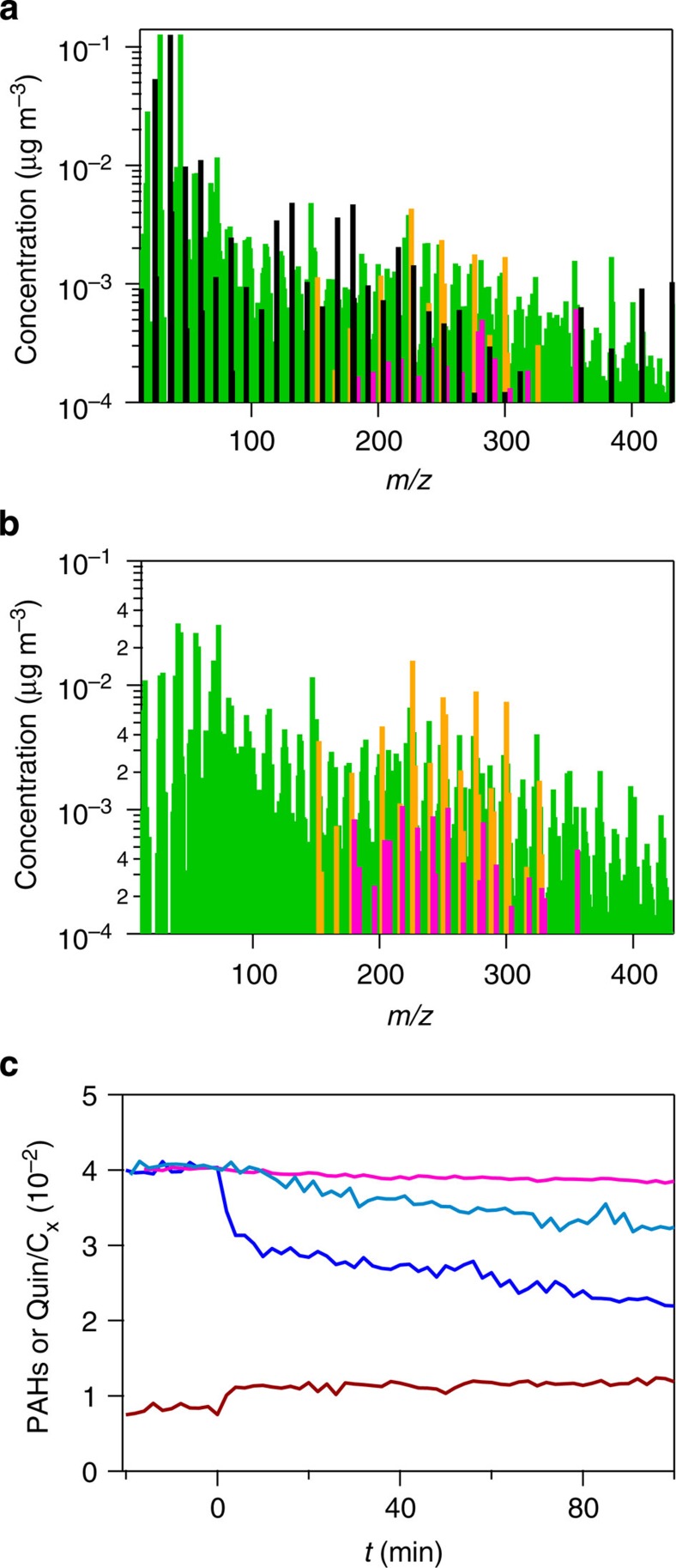
Polycyclic aromatic hydrocarbons (PAHs) on uncoated soot particles. (**a**) Typical SP–AMS (soot-particle aerosol mass spectrometer) spectrum (obtained with the laser vaporizer) of organic species (green) and elemental carbon species (C_*x*_, black) in soot particles used in this work. Highlighted in orange are the species considered to be PAHs and in pink those considered to be quinones. (**b**) AMS (aerosol mass spectrometer) spectrum (obtained with the thermal vaporizer) for the soot, (**c**) Typical time evolution of the SP–AMS signals normalized to elemental carbon of PAHs at 1.5 × 10^12^ molecule cm^−3^ O_3_ (pink), at 3.4 × 10^12^ molecule cm^−3^ O_3_ (light blue) and at 1.1 × 10^13^ molecule cm^−3^ (dark blue), and quinones at 1.1 × 10^13^ molecule cm^−3^ O_3_ (red); *t*=0 min is the time at which O_3_ was added to the chamber.

To monitor the composition of the particles, measurements with a recently developed instrument, the Soot–Particle Aerosol Mass Spectrometer (SP-AMS)^29^,^30^, were performed. The SP–AMS detects refractory black carbon (rBC) and associated non-refractory species via a two-step process involving, first, laser vaporization and, second, electron impact ionization. In this manner, most species detected by the SP–AMS were elemental carbon, detected as C_*x*_ ions where *x*≥1, and organic molecules, appearing as both molecular ions and organic fragment ions. Some experiments were done having the thermal vaporizer removed from the SP–AMS, so only particles containing rBC could be detected. This confirmed that the organic species seen in the spectra were internally mixed with rBC. The mass spectrum of bare soot is shown in Fig. 1a, where only organic and elemental carbon mass-to-charge ratios are included. Although other species such as nitrate, sulfate and ammonium were also detected, they have not been included in this spectrum as they are negligible. Using standard relative ionization efficiencies for these two species (see Methods), the ratio of the mass of organic species to the mass of elemental carbon species was 0.59±0.12.

High resolution mass spectral analysis was performed to identify molecular ions of PAHs in the spectrum, as shown in Fig. 1a, that is, these peaks can only arise from unsaturated C_*x*_H_*y*_ species. PAHs are a commonly identified constituent class within AMS spectra and very likely to be present in soot particles. Confidence that these peaks arise from PAHs is enhanced through the measurements of kinetics to be described below. PAH molecular ions (general formula C_*x*_H_*y*_, with *x*>*y*) were chosen because PAHs are not highly fragmented by electron impact ionization. The species highlighted in Fig. 1a were selected based on the PAHs that were measured on ambient particles in Mexico City using aerosol mass spectrometry^27^ and are listed in Supplementary Table 2. This is probably a subset of all PAHs in the soot, given that other species are likely present including alkylated species that will fragment to a larger degree under electron impact conditions. The chosen PAHs are generally large PAHs that partition principally to particles. On average, the ratio of the mass of these PAHs to the mass of elemental carbon in our soot particles was 0.04±0.02.

Also shown in Fig. 1a are the ions that are likely arising from quinones that are structurally similar to the PAHs under consideration. Quinones fragment more than PAHs, so besides the molecular ions, M^·+^ (C_*x*_H_*y*−2_O_2_^·+^), M-CO^·+^ (C_*x*−1_H_*y*−2_O^·+^) ions were considered as markers for these species. This fragmentation pattern was confirmed by SP–AMS observation of the oxidation of adsorbed benzo[*e*]pyrene, in the experiments described below. We cannot rule out that some of the peaks that have been attributed to quinones have contributions due to other species or mass spectral fragments with the same molecular formula.

The time evolution of the signals attributed to total PAHs and quinones is plotted in Fig. 1c. The signals were normalized to the total elemental carbon signal to take into account particle loss during the course of the experiment. It can be seen that the signal attributed to PAHs decreased when soot particles were exposed to O_3_, whereas the signal corresponding to quinones simultaneously increased. Decays of PAHs are shown in Fig. 1c at three O_3_ concentrations: 1.5 × 10^12^, 3.4 × 10^12^ and 1.1 × 10^13^ molecule cm^−3^, with slower decays observed at lower O_3_ levels.

A standard AMS spectrum (that is, obtained by using only the thermal vaporizer, with the rBC volatilization laser off) for non-refractory species coated on soot particles is shown in Fig. 1b. Molecular ions for potential PAHs were also detected in the spectra, in many cases rising above the organic background in a more pronounced manner than in the SP-AMS spectrum where the whole particle is volatilized. This might indicate that, along with other organics, PAHs are preferentially on the surface of soot, whereas other organics (and presumably some PAHs too) are buried within soot and are only detected when the particle is fully volatilized by the laser of the SP–AMS. Ions for potential quinones were also detected in the AMS spectrum, indicating that these species can also be located on the surface of soot particles.

For those experiments in which soot particles were coated with BeP, the peak at *m/z* 252 that corresponds to the BeP molecular ion (C_20_H_12_^·+^) was one of the most intense peaks in the SP–AMS spectrum (see Supplementary Fig. 1a). Other peaks of high intensity include fragments of ionized BeP. The time evolution of the peak at *m/z* 252 depicted in Supplementary Fig. 1b illustrates a clear decrease of the BeP signal when O_3_ is added to the chamber. At the same time, the peak at *m/z* 282 that corresponds to the molecular formula of the BeP quinone ion, C_20_H_10_O_2_^+^, rises in intensity. In this manner, the experiments with BeP-coated soot confirm that particle-bound PAHs are oxidized under exposure to ozone in the reaction chamber, and that among likely products are the corresponding particle-bound quinones.

To support these observations, diffuse reflectance infrared fourier transform (DRIFT) spectra were recorded to observe functional group changes occurring on the surface of soot particles when they were exposed to ozone. In Supplementary Fig. 2, spectra are included for bare soot before oxidation (where the reference spectrum is that of KBr powder) and soot after 15 and 360 min of exposure to ozone at 4.9 × 10^14^ molecule cm^−3^ (where the reference spectrum is that of the un-oxidized soot mixed into KBr powder). These spectra were recorded in experiments done in the reaction chamber of the DRIFT set-up. Although not shown here because of their lower quality, the spectra recorded for soot taken from the chamber exhibited the same results.

Before oxidation, soot exhibits three regions of interest in its IR spectrum: a band centred at 1,600 cm^−1^ that can be attributed to the stretching of C=O bond in carbonyl groups of quinone-like species, as Sutherland *et al.*^31^ suggested for carbon blacks and Gulyás *et al.*^32^ for carbon fibres, another band centred at 1,250 cm^−1^ that is due to the stretching of C-O in various chemical environments, also observed by Sutherland *et al.*^31^, and a group of small peaks, very much influenced by the noise from the detector, between 940 and 550 cm^−1^ that could correspond to the C-H bending mode of aromatic groups.

When the soot was exposed to O_3_, the spectra show clear indication of oxidation. In particular, there is a small increase of the 1,600 and 1,250 cm^−1^ bands observed, and the appearance of bands centred at 1,700, 1,370 and 1,000 cm^−1^. The 1,700-cm^−1^ band is typical of C=O bond stretching in ketones or carboxylic acids. In particular, Sutherland *et al.*^31^ also observed by transmission FT-IR the appearance of a band at around 1,700 cm^−1^ when they oxidized carbon blacks with O_3_. They found that the absorbance of that band was related to the surface oxygen concentration measured by X-ray photoelectron spectroscopy. Holder *et al.*^8^ oxidized methane soot with a mixture of O_3_ and NO_*x*_, and they also detected the increase of the band at 1,700 cm^−1^, assigned to carbonyl groups. We believe the increase of the 1,600 cm^−1^ band is likely due to the increase of quinone-like functional groups on the surface of the particles, and the 1,370 and 1,000 cm^−1^ bands are probably due to C-O bonds. DRIFT spectra for soot coated with BeP showed no observable difference when compared with the spectra obtained for bare soot, due to the thin coatings used.

### Uptake coefficient of O_3_ on PAHs linked to soot particles

Decays of the PAH or BeP signal from O_3_ exposure to soot particles (Fig. 1c and Supplementary Fig. 1b) were fitted to a double-exponential equation. This double time constant behaviour was previously observed during the photodegradation of PAHs^33^. The loss may be attributed to a mechanism in which near-surface-bound PAHs react with O_3_ and are depleted in the first stage of reaction, and, on a slower timescale, losses of PAHs also arise from diffusion within the particle. Two pseudo-first order rate constants were obtained for each decay: *k*_1_′, which corresponds to the fast loss that is observed at the beginning of each decay, and *k*_2_′, which describes the slow loss during the latter part of each decay. In the subsequent analysis only *k*_1_′ is considered, as it corresponds to the dominant process. Results for *k*_2_′ are presented in Supplementary Fig. 3.

**Figure 2:**
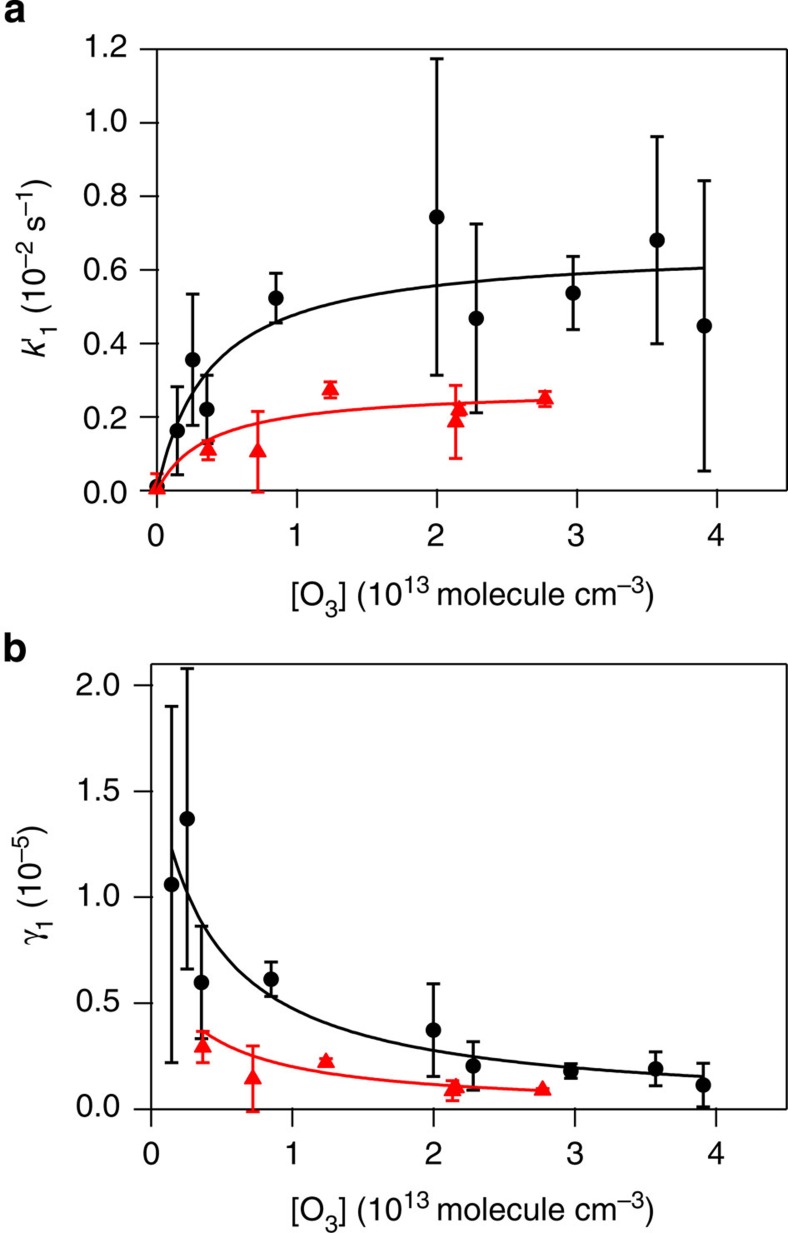
Kinetic results for uncoated and benzo[*e*]pyrene (BeP)-coated soot. (**a**) Pseudo-first order rate constants for the fast loss process (1) observed in PAH (polycyclic aromatic hydrocarbon) decay as a function of O_3_ concentration for the reactions with total PAHs on soot (black circles) and BeP on soot (red triangles). Data were fitted to equation 1. (**b**) Uptake coefficients calculated from the data in the upper graph using equation 2 as a function of O_3_ concentration. Data were fitted to equation 3. Every point corresponds to one experiment. Error bars represent one standard deviation.

An enhancement of *k*_1_′ was observed when the O_3_ concentration increased, as plotted in Fig. 2a. This enhancement became less important at high O_3_ concentration until *k*_1_′ reached a maximum value, which is consistent with the Langmuir-Hinshelwood mechanism as previously reported for the reaction of BaP and O_3_ (refs 23, 24). A reaction via this mechanism involves a species, such as the total PAHs or BeP, that is strongly adsorbed to the particle surface and a gas-phase species, O_3_, which is at equilibrium with the gas phase and the particle surface. The reaction is a two-step process, with the adsorption of O_3_ followed by a surface reaction. According to this model, the number of sites that are available for the reaction with O_3_ is limited so that when a certain O_3_ concentration is reached, the surface is saturated. The data shown in Fig. 2a were fitted to the Langmuir–Hinshelwood equation^24^:


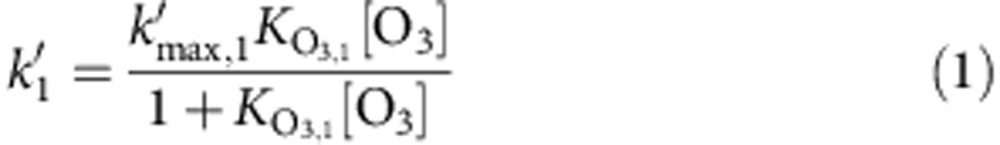


where 
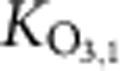
 is the ozone gas-to-surface equilibrium constant and *k*′_max,1_ is the maximum rate coefficient that would be observed at high ozone concentration. The subscript 1 indicates that these constants refer to the fast loss observed at the beginning of the double exponential decay. 
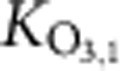
 and *k*′_max,1_ were determined from this fitting. For the slower loss observed in the decay, as it is due to a diffusive process that may not be described by Langmuir–Hinshelwood kinetics, we do not fit the data in Supplementary Fig. 3a to this mechanism.

The experimental 
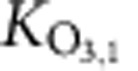
 values determined in this work are very similar for total PAHs and BeP on soot: (2.6±1.6) × 10^−13^ and (2.7±1.3) × 10^−13^ cm^3^, respectively, similar to that previously reported for BaP on soot: (2.8±0.2) × 10^−13^ cm^3^ (ref. 23). This indicates that for all these processes, O_3_ is partitioned between the soot surface and the gas phase to a similar extent. The values of *k*′_max,1_ obtained in this study differ somewhat for total PAHs and BeP ((6.5±3.1) × 10^−3^ s^−1^ and (2.7±1.1) × 10^−3^ s^−1^, respectively) and the previously reported value ((1.5±0.1) × 10^−2^ s^−1^) for BaP^23^. These results indicate that BaP is more reactive than BeP, as already reported by Perraudin *et al.*^25^ *k*′_max,1_ for total PAHs corresponds to a mixture of the rate constants of the different PAHs that are on soot particles.

For a bimolecular reaction on the surface, the uptake coefficient is calculated with the following equation:


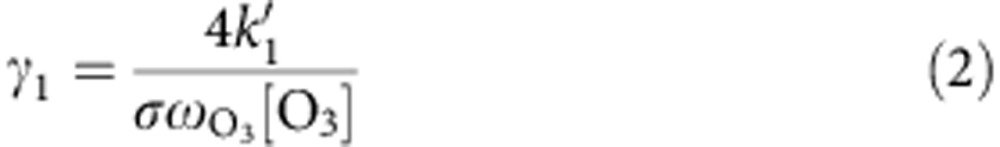


where *σ* is the molecular cross-section of a PAH, that was considered to be the same as for BaP, 10^−14^ cm^2^ (refs 23, 24), and 
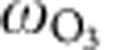
 is the mean velocity of O_3_. The resulting *γ*_1_ values are on the order of 10^−5^–10^−6^ for total PAHs and 10^−6^ for BeP on soot. The uptake coefficients determined in this work for the first phase of the decay are plotted as a function of O_3_ concentration in Fig. 2b, and with the solid line determined by equation 3, which is the result of combining equations 1 and 2.


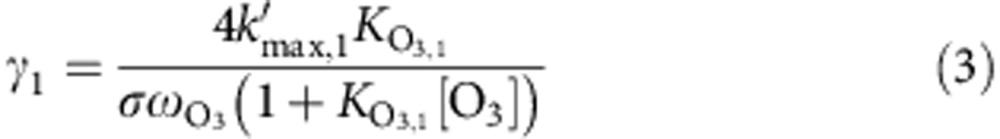


### Changes in the soot redox cycling activity due to oxidation

To measure the redox cycling rates of the soot particles, we employ the DTT assay^26^ where dithiothreitol (DTT) acts as the reducing agent in place of biological molecules, such as glutathione and ascorbate. Despite it being an indirect measure of one aspect of toxicity, several studies have used the DTT assay to evaluate the toxicity of different chemical species^7^,^8^,^9^,^26^,^34^,^35^,^36^,^37^,^38^.

**Figure 3:**
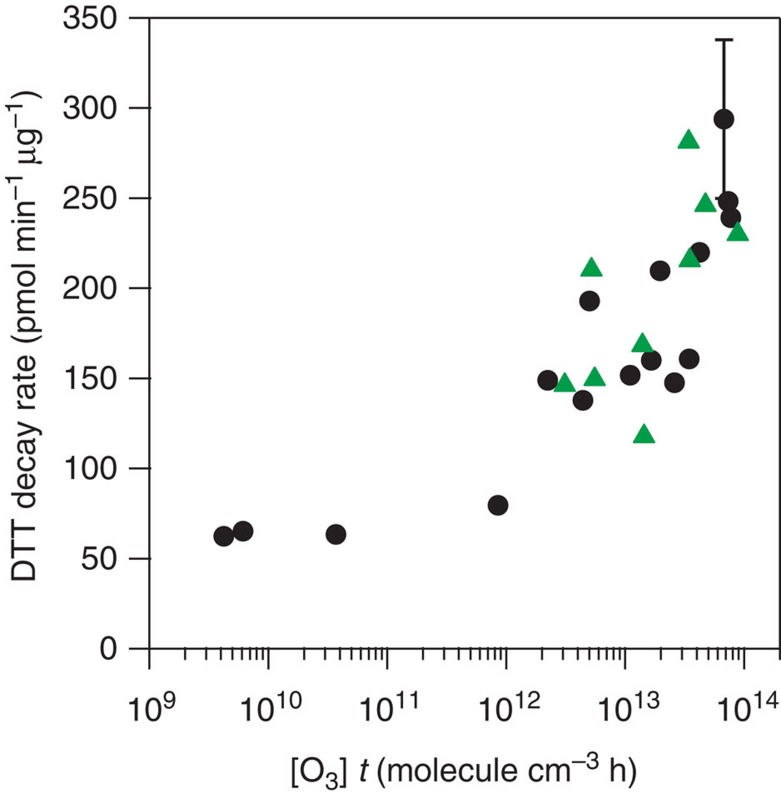
Redox cycling results. Mass-normalized DTT (dithiothreitol) decay rate measured for bare soot (black circles) and soot coated with BeP (green triangles) as a function of O_3_ exposure. A systematic error bar of ±15% for the DTT decay rate was included for one point.

The mass-normalized DTT decay rates of oxidized particles are shown as a function of O_3_ exposure ([O_3_]*t*) in Fig. 3 for both bare soot and BeP-coated soot. In all cases the data plotted are the total DTT activity, without filtering the buffer extract solutions prior to analysis. We estimate a ±15% systematic uncertainty as typical for each DTT decay point in the figure that incorporates the uncertainties in the mass of soot measured on the filter and from the statistical analysis of the decay rates. An additional variability (on the order of ±40%) sometimes arises due to the assay response itself when conducted with soot, as compared with the assay with a fully soluble species. In particular, the soot mixtures were quite heterogeneous in nature with different degrees of coagulation, thus affecting their response.

Before oxidation, the redox activity of bare soot was 49±7 pmol min^−1^ μg^−1^. Figure 3 demonstrates that at O_3_ exposures lower than 10^12^ molecule cm^−3^ h, the increase in DTT activity is very low compared with what is observed at higher exposures. This indicates that the number of reactive encounters of ozone at lower exposures is not sufficient to significantly oxidatively process the surface of soot. However, at higher exposures, it is possible to link the kinetics of PAHs on soot to the activation of the DDT response of soot. In particular, the concentration of O_3_ used for the experiment at which the O_3_ exposure was ∼10^12^ molecule cm^−3^ h was 3 × 10^12^ molecule cm^−3^ (see Supplementary Table 3). At this concentration, the rate coefficient *k*_1_′ is ∼2 × 10^−3^ s^−1^ (see Fig. 2a), corresponding to a time constant for the loss of PAHs of ∼10 min. This is in excellent agreement with the 15 min of time required to generate the exposure of ∼10^12^ molecule cm^−3 ^h that leads to the increase in the DTT activity in Fig. 3. Moreover, the decays present in Fig. 1c corroborate that, at low O_3_ levels, the oxidation of PAHs is minor. The fact that the DTT activity increases at an O_3_ exposure that matches the kinetics for the oxidation of a PAH with O_3_ indicates that the oxidation of PAHs (or PAH-like structures) present on soot particles, and the formation of their oxidation products, is responsible for the increased redox activity of these particles. Among the oxidation products that contribute to the increase of the redox activity are quinones, although it is likely that these are not the only oxidation products that contribute to the DTT activity. In additional experiments, we removed quinones present in soot by the derivatization process that has been widely used in quinone analysis^39^. When the DTT assay was performed on the samples that were derivatized, the decrease of the redox activity was 76% for the non-oxidized soot, and 45% for the oxidized soot, indicating the possible contribution of other species than quinones to the increase in the redox activity.

DTT activity keeps increasing at high O_3_ exposures, whereas *k*_1_′ levels off at [O_3_]∼10^13^ molecule cm^−3^. The additional DTT activity either arises from the oxidation of the buried PAHs or else it arises from more redox active species formed by subsequent oxidation of the first generation products. Indeed, in previous work from our laboratory involving the redox cycling activity of naphthalene secondary organic aerosol, we observed that a multiple generation oxidation product, a hydroxylated napthaquinone, contributed significantly to the overall redox cycling activity of the particles formed^40^.

Our redox cycling rates are in qualitative agreement with previous studies. In particular, Holder *et al.*^8^ observed an increase in the DTT activity when they oxidized methane soot at very high O_3_ concentrations in the presence of NO_*x*_. Li *et al.*^9^ exposed DEP to lower levels of O_3_ and NO_*x*_, and also observed an increase of the DTT activity. McWhinney *et al.*^7^ found an increase in redox cycling rates when combustion particles were exposed to different O_3_ concentrations. However, they attributed this behaviour to the presence of SOA forming on the surface of the particles due to the reaction of engine emission gases and O_3_. Rattanavaraha *et al.*^10^ also detected an increase of the redox activity when DEP were exposed to sunlight in an outdoor chamber, in both the presence and absence of a volatile hydrocarbon mixture. In our work, we have a less complicated mixture of gases due to the nature of the fuel, and denuders were used to remove possible interferences from gas-phase species. Therefore, the formation of SOA in our system is less important, with the ratio of the mass of organic to elemental carbon changing by <15% after oxidation.

A negligible difference between the DTT decay rate for bare soot and soot coated with BeP was observed, which is consistent with PAHs not being redox active. For the oxidized particles, it is not possible to say within the uncertainties whether an increase in activity was observed when BeP is present. Although an increase of redox activity could be expected, it is possible that the redox activity of BeP oxidation products was similar to the activity of bare oxidized soot, yielding no change between uncoated soot and soot coated with BeP. We note that DTT decay rates obtained in this work for oxidized soot are roughly an order of magnitude higher than those determined for ambient PM^26^,^34^,^35^,^36^,^37^, indicating the potential significance for aged soot particles to contribute to the overall redox cycling abilities of ambient PM.

## Discussion

This study provides the first mechanistic connection between the increased redox cycling abilities of oxidized soot particles and their chemical composition. In particular, novel aerosol mass spectrometry measurements indicate that PAH-like species within the soot particles decay on ozone exposure, with a corresponding increase in the mass spectrometric signals attributed to quinones. The formation of surface-bound quinone-like species is confirmed by infrared analysis. These findings were corroborated by oxidation experiments performed with soot particles coated with BeP, in which BeP clearly decayed while the mass spectrometric peak of its corresponding quinone increased. The capability of the oxidized particles to be catalysts of electron-transfer processes increased, consistent with quinones being good redox cycling agents.

The increases of the redox cycling activity of combustion particles when exposed to O_3_ were conducted in a low-NO_*x*_ and low-volatile organic compound (VOC) environment. Although in the ambient atmosphere levels of NO_*x*_ and VOCs are much higher, the results obtained in this work isolate the effects of heterogeneous ozone oxidation on the soot surface and the corresponding changes in redox cycling ability. To measure a significant change in the redox cycling rate, an ozone exposure equivalent to 3 h at 7.4 × 10^11^ molecule cm^−3^ (30 p.p.b. by volume), a typical background level^41^, was required. In more polluted environments, where soot particles are more likely to be emitted, and where O_3_ mixing ratios can reach over 2.5 × 10^12^ molecule cm^−3^ (100 p.p.b. by volume)^41^, the redox cycling activity would reach its maximum value in less time.

## Methods

### Reaction chamber set-up

Experiments were performed in batch mode in a 1-m^3^ environmental Teflon bag full of clean air. Soot particles were generated by a soot generator (miniCAST 6203A, Jing) that uses a propane-air diffusion flame. A flow of particles diluted in air (6 slpm, standard liter per minute) was emitted by the soot generator, but only 1 slpm was passed through a denuder filled with Carulite 200 (MnO_2_/CuO catalyst, Carus Corporation) before entering the chamber. This denuder is usually used to remove O_3_ in the gas phase but it also decreased by 98% the levels of NO and NO_2_ generated in the combustion process. In some experiments, an activated charcoal (Norit PK 3–5, Sigma-Aldrich) denuder was used to remove VOCs, although no changes in the results were observed when it was employed.

For the experiments in which the soot particles were coated by BeP, the flow of particles was also passed through a glass tube (*l*=35 cm, *d*=1 cm) with the inner walls covered by BeP and heated using external heating tapes to 375 K. To cover the walls of the tube, BeP was dissolved in CH_2_Cl_2_ and the solution was spread on the inner walls of the tube by rotating and inclining it while the solvent was evaporating. These conditions made possible an estimated average BeP coating thickness of 3 × 10^−2^ nm on the soot particles, that is, well less than that of a monolayer. This estimation was done following the same method that Zhou *et al.*^42^ followed in which assumes a monodisperse size distribution of particles, a uniform coating thickness of the BeP over the particles, an assumption that the particle surface area is that of a sphere with diameter set by its mobility diameter and the amounts of BeP as measured by aerosol mass spectrometry (see below). Given that the surface area of soot will be far higher than that of a smooth sphere, we are quite confident that the coatings of BeP are very thin, and that the soot is likely not fully coated by the PAH.

Once soot particles were in the chamber, O_3_ was introduced at concentrations ranging 1.75 × 10^12^ and 1 × 10^14^ molecule cm^−3^ (70–4,000 p.p.b.). It was generated by passing 1 lpm of air (Linde, Grade 0.1) or O_2_ (Linde, Grade 2.6) over a 185 nm Hg pen-ray lamp (UVP). Five gas analysers were used to measure the mixing ratios inside the chamber of O_3_ (Model 49*i*, Thermo Scientific), NO_*x*_ (=NO+NO_2_) (Model 42*i*, Thermo Scientific), SO_2_ (Model 43*i*, Thermo Scientific), CO (Model 48*i*, Thermo Scientific) and CO_2_ (Model 410*i*, Thermo Scientific). The number densities of soot particles and their size distributions were determined by a SMPS consisting of a differential mobility analyser (DMA, TSI) to size-select the particles and a condensation particle counter (CPC, TSI) to measure their number concentration. Flow rates going to the gas analysers and SMPS were 3.5 slpm and 0.3 slpm, respectively.

### Soot-particle aerosol mass spectrometer

A SP–AMS (Aerodyne Research; more details in Onasch *et al.*^29^) was used to observe chemical changes in soot particles when they were exposed to O_3_. The SP–AMS is a high resolution time-of-flight aerosol mass spectrometer (HR-ToF-AMS) with an intracavity Nd:YAG laser vaporizer (*λ*=1,064 nm). In a standard HR-ToF-AMS, a beam of particles is first generated with an aerodynamic lens and then flash vaporized on a heated tungsten surface. The vapours are ionized by electron impact, and the resulting ions are detected with high mass resolution in a time-of-flight mass spectrometer. The tungsten furnace (∼600 °C) only vaporizes non-refractory material, whereas the laser vaporizer allows the detection of refractory black carbon particles, and non-refractory materials that are internally mixed with soot. Data were analysed using SQUIRREL (v1.51H) and its corresponding module for high-resolution data, PIKA (v1.10H), running within Igor Pro (v6.22A, WaveMetrics). The SP–AMS was calibrated with 300 nm Regal Black particles (Regal 400R, Cabot Corp.) and with NH_4_NO_3_ as described by Onasch *et al.*^28^ Standard relative ionization efficiencies of 0.2 and 1.4 were used for elemental carbon species and organic species, respectively^29^,^43^.

### Collection of filters and DRIFTS

Once the exposure to ozone was complete, the particles in the chamber were pulled onto a polytetrafluoroethylene (PTFE) filter (Pall Life Sciences, 2 μm pore size, 47 mm diameter). Ozone exposure was changed by modifying either time or [O_3_]. Some of the samples collected on the filters were used to further characterize the chemical composition of the particles by DRIFT spectroscopy (DRIFTS). A FT–IR spectrometer (Magna-IR 550 Series II, Nicolet) accumulated 1,000 scans per spectrum at a resolution of 2 cm^−1^, and a diffusion reflection accessory (Praying Mantis, Harrick) was located in the sample compartment to measure DRIFT spectra. Reference spectra using dried KBr powder (<10 μm particle size, FT-IR grade, Sigma-Aldrich) were always recorded before spectra of the samples were measured, and samples were always diluted by adding KBr until the content of soot was around 0.2% of the total mass. Complementary experiments of soot oxidation were done in a reaction chamber inside the sample compartment of the diffuse reflection accessory (Praying Mantis diffuse reflection environmental chamber) to better characterize the DRIFT spectrum. A 100 sccm (standard cubic centimetre per minute) flow of 20 p.p.m. O_3_ was passed through the reaction chamber, and spectra were recorded

### DTT assay

Particles collected on the filters were also used to measure the redox cycling activity of the particles immediately after collection. Filters were placed in 10 ml phosphate buffer solution (0.1 M, pH 7.4) and sonicated for 15 min to obtain suspensions of soot that underwent the DTT assay^26^ after an appropriate dilution (soot concentration in the diluted samples ranged between 1.2 and 14 μg ml^−1^). The extraction efficiency from the filter to the buffer solution was observed to be around 80%. Control experiments were done sonicating clean filters to corroborate that the influence on the DTT response of peroxides generated in this process is negligible. For some of these suspensions, aliquots were filtered (PTFE VWR syringe filters, 0.2 μm pore size, 13 mm diameter) before measuring their DTT activity to determine the activity due to water-soluble species only. The assay of the soot suspension measured the redox activity due to both soluble species and species present at the surface of the soot particles. For the assay, 1 ml aliquots of sample were combined with 0.25 ml of 0.5 mM DTT in pH 7.4 phosphate buffer, and kept at 310 K to simulate human-body temperature. The reaction was quenched over the course of 1 h by adding 0.25 ml of the reaction mixture to 1 ml of 5,5′-dithiobis-(2-nitrobenzoic acid) (DTNB). A yellow-coloured compound (2-nitro-5-thiobenzoate) was formed, and its absorption was measured at 412 nm. The DTT decay rate of a blank solution was subtracted every time the assay was performed, and the results were mass-normalized to make them comparable from sample to sample. In Supplementary Fig. 4, examples of the decay of DTT for non-filtered solutions are shown at three different exposures to O_3_ together with the decay observed for a quinone that is known to be active in the DTT assay, 1,4-naphthoquinone (DTT decay rate=3,100±280 pmol min^−1^ μg^−1^). The measurements of the DTT activity were repeated for one solution in different days, and reproducible results were observed, showing a negligible effect of partitioning between particle and aqueous phase.

## Author contributions

M.A., S.Z. and J.A. conceived and designed the experiments; M.A., M.W. and S.Z. performed the experiments; M.A. and M.W. analysed the data; M.A., M.W., S.Z. and J.A. co-wrote the paper.

## Additional information

**How to cite this article:** Antiñolo, M. *et al.* Connecting the oxidation of soot to its redox cycling abilities. *Nat. Commun.* 6:6812 doi: 10.1038/ncomms7812 (2015).

## Supplementary Material

Supplementary Information Supplementary Figures 1-4 and Supplementary Tables 1-3
