# Supplementary material for: Connecting the oxidation of soot to its redox cycling abilities
Source: Nat Commun. 2015 Apr 15;6:6812. doi: 10.1038/ncomms7812 (PMC4410628; doi:10.1038/ncomms7812)
Supplement: Supplementary Information — Supplementary Figures 1-4 and Supplementary Tables 1-3 [file ncomms7812-s1.pdf]

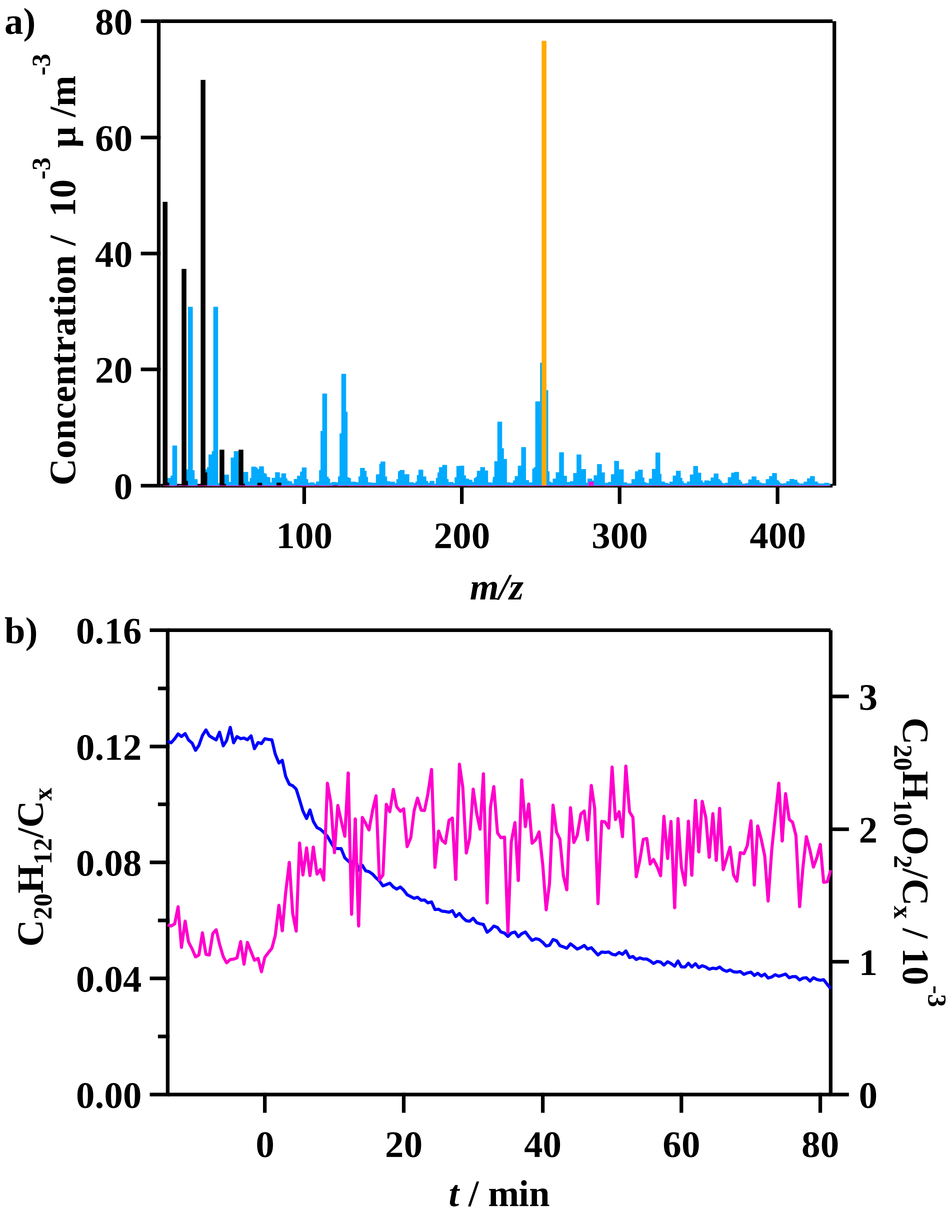

Supplementary Figure 1. Polycyclic aromatic hydrocarbons (PAHs) on benzo[*e*]pyrene (BeP) coated soot particles. a) Example of SP-AMS (Soot-Particle Aerosol Mass Spectrometer) spectrum of soot particles coated with BeP. Organic species are depicted in blue, whereas carbon species are in black. The orange line at *m/z* = 252 corresponds to the BeP molecular ion ($C_{20}H_{12}^{+}$). b) Time evolution of the signals normalized to carbon of BeP (blue) and its corresponding quinone (pink). *t* = 0 min indicates the time at which O_3 was added to the chamber in that experiment.

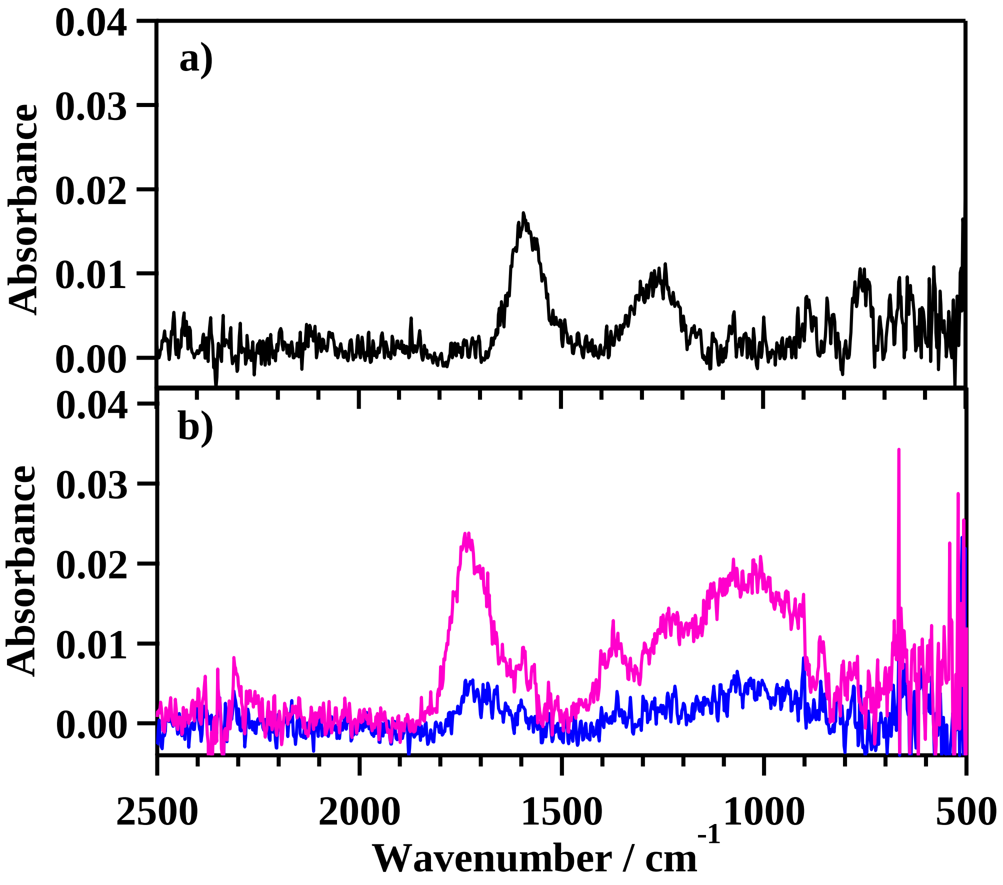

Supplementary Figure 2. DRIFT (Diffuse Reflectance Infrared Fourier Transform) spectra for bare soot. (a) Spectrum before O_3 exposure to 20 ppm (parts per million) O_3 and when the reference spectrum was KBr. (b) Spectra after exposure to 20 ppm, when the reference spectrum as unoxidized soot and KBr (see text). Time of exposure to O_3: 0 min (black), 15 min (blue), and 360 min (pink).

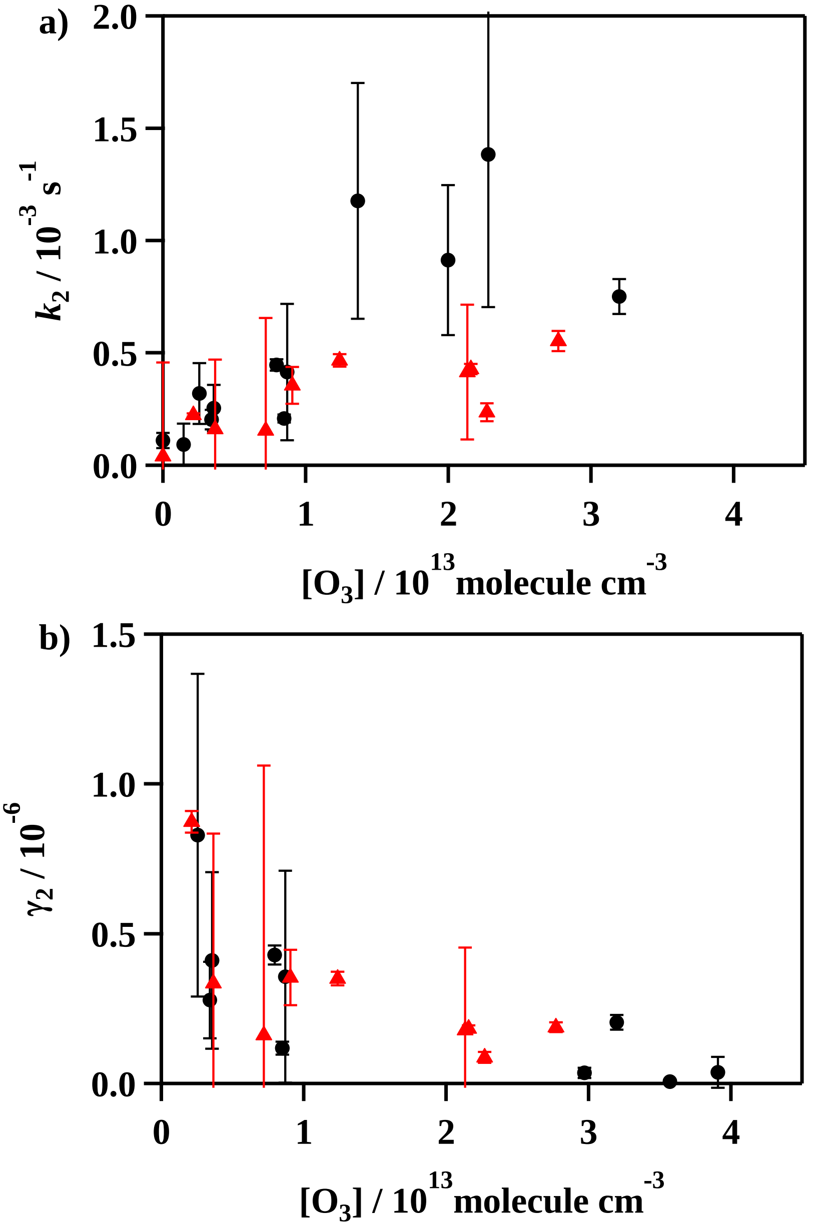

Supplementary Figure 3. Kinetic results for uncoated and benzo[*e*]pyrene (BeP) coated soot. a) Pseudo-first order rate constants for the slow loss process observed in the decays as a function of O_3 concentration for the reactions with total PAHs (Polycyclic Aromatic Hydrocarbosn) in soot (black) and BeP in soot (red). b) Uptake coefficients calculated from k_2' using equation 2. Every point corresponds to one experiment. Error bars represent standard deviation.

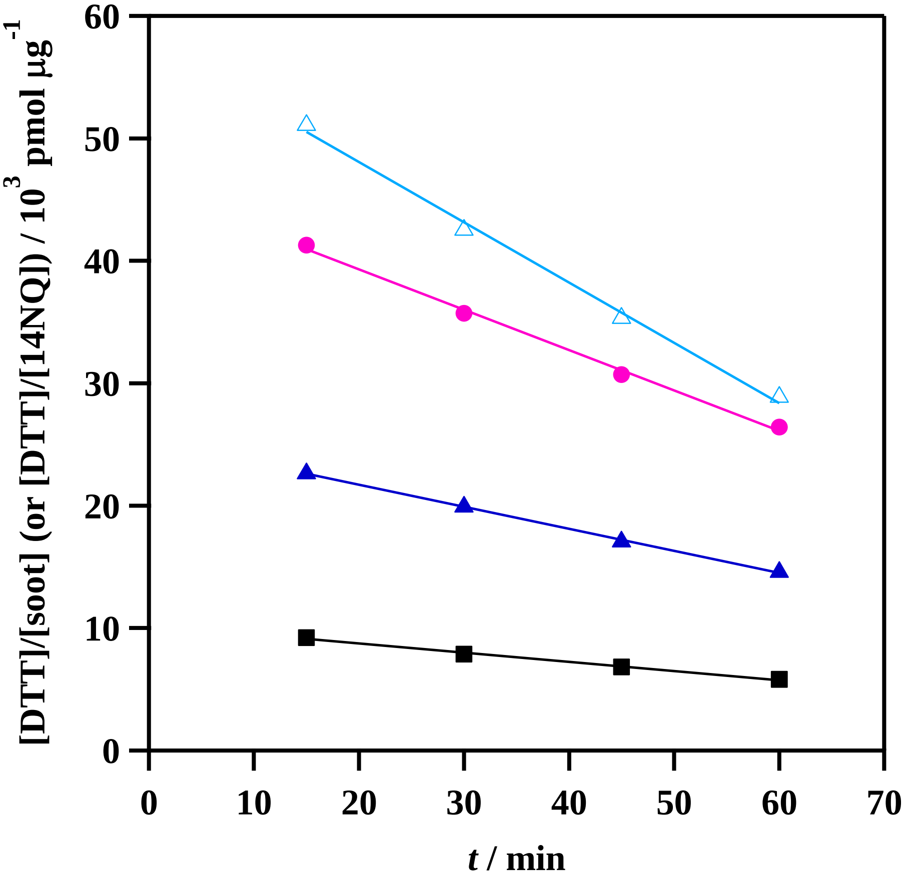

Supplementary Figure 4. Decay of dithiothreitol (DTT) concentration. Sample decays of DTT concentration as a function of time for 1,4-naphthoquinone (14NQ, light blue triangles) and soot at three different O_3 exposure times (no exposure, in black squares; 6×10^9 molecule cm^{-3} h in blue triangles; and 4×10^{13} molecule cm^{-3} h in pink circles). [DTT] (i.e. moles per volume) was normalized with the concentration of soot or quinone (i.e. mass per volume) that was used to measure the decay rate. Lines represent the linear fitting of the points.

Supplementary Table 1. Experimental conditions. Average experimental conditions inside the smog chamber at the start of the experiments for the DTT (dithiothreitol) assay. Experiments for SP-AMS (Soot-Particle Aerosol Mass Spectrometer) analysis were performed with lower soot mass loadings.

Measurement	Value
**Mean diameter of particles of the number distribution**	327 nm
**Effective density**	0.2 g cm^{-3}
**Particle concentration**	1.5×10^{5} cm^{-3}
**Mass loading**	6.7 mg m^{-3}
**[O_3]**	70-4000 ppb *
**[NO]**	9 ppb *
**[NO_x]**	22 ppb *
**[SO_2]**	1 ppb *
**[CO_2]**	206 ppm **
**[CO]**	1120 ppb *

*ppb: parts per billion, ** ppm: parts per million

Supplementary Table 2. PAHs (polycyclic aromatic hydrocarbons) list. PAHs that were considered in the analysis of the SP-AMS (Soot-Particle Aerosol Mass Spectrometer) data.

Molecular Formula	*MW* / g mol^{-1}	Structure	Name
$C_{12}H_8$	152	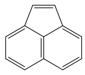	Acenaphthylene
$C_{12}H_{10}$	154	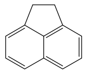	Acenaphthene
$C_{13}H_{10}$	166	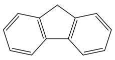	Fluorene
$C_{14}H_{10}$	178	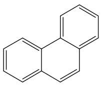	Phenanthrene
$C_{14}H_{10}$	178	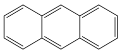	Anthracene
$C_{16}H_{10}$	202	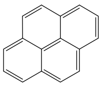	Pyrene
$C_{16}H_{10}$	202	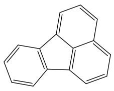	Fluoranthene
$C_{16}H_{10}$	202	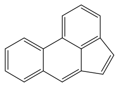	Acephenanthrylene
$C_{17}H_{12}$	216	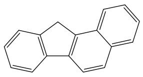	1,2-Benzofluorene
$C_{17}H_{12}$	216	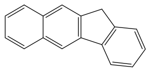	2,3-Benzofluorene
$C_{17}H_{12}$	216	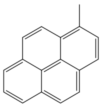	1-Methylpyrene
$C_{18}H_{10}$	226	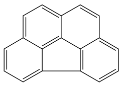	Benzo[*ghi*]fluoranthene

Molecular Formula	*MW* / g mol^{-1}	Structure	Name
$C_{18}H_{10}$	226	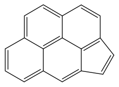	Cyclopenta[*cd*]pyrene
$C_{18}H_{12}$	228	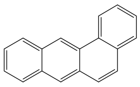	Benz[*a*]anthracene
$C_{18}H_{12}$	228	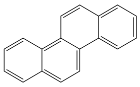	Chrysene
$C_{18}H_{12}$	228	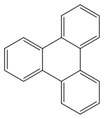	Triphenylene
$C_{19}H_{12}$	240	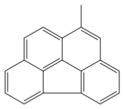	Methylbenzo[*ghi*]fluoranthene
$C_{19}H_{14}$	242	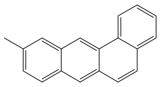	10-Methylbenz[*a*]anthracene
$C_{20}H_{10}$	250	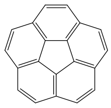	Corannulene
$C_{20}H_{10}$	250	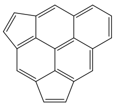	Dicyclopental[*cd,mn*]pyrene
$C_{20}H_{12}$	252	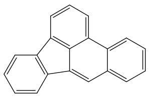	Benzo[*b*]fluoranthene
$C_{20}H_{12}$	252	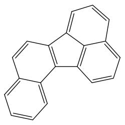	Benzo[*j*]fluoranthene
$C_{20}H_{12}$	252	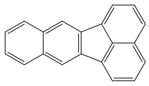	Benzo[*k*]fluoranthene
$C_{20}H_{12}$	252	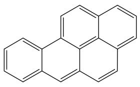	Benzo[*a*]pyrene
$C_{20}H_{12}$	252	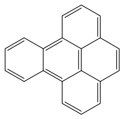	Benzo[*e*]pyrene

Molecular Formula	*MW* / g mol^{-1}	Structure	Name
$C_{21}H_{12}$	264	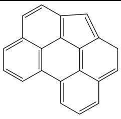	11*H*-Cyclopenta[*ghi*]perylene
$C_{21}H_{14}$	266	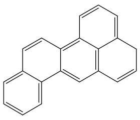	4*H*-Benzo[*hi*]chrysene
$C_{22}H_{12}$	276	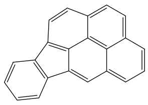	Indeno[1,2,3-*cd*]pyrene
$C_{22}H_{12}$	276	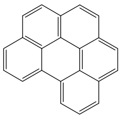	Benzo[*ghi*]perylene
$C_{23}H_{12}$	288	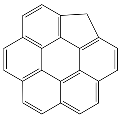	1*H*-Benzo[*ghi*]cyclopenta[*pqr*]perylene
$C_{23}H_{14}$	290	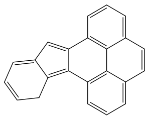	9*H*-Indeno[1,2-*e*]pyrene
$C_{24}H_{12}$	300	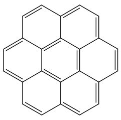	Coronene
$C_{24}H_{14}$	302	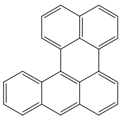	Benzo[*a*]perylene
$C_{24}H_{14}$	302	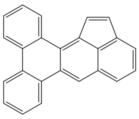	Indeno[1,7-*ab*]triphenylene
$C_{25}H_{16}$	316	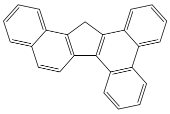	15*H*-Benz[4,5]indeno[1,2-*l*]phenanthrene
$C_{26}H_{14}$	326	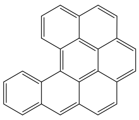	Dibenzo[*a*,*ghi*]perylene
$C_{26}H_{14}$	328	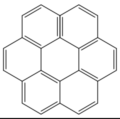	Phenanthro[3,4-*c*]phenanthrene

Supplementary Table 3. O_3 exposures. Time and average O_3 concentration used in the oxidation of bare soot particles at each reported O_3 exposure.

$[O_3]t$ / 10^{12} molecule cm^{-3} h	$[O_3]$ / 10^{12} molecule cm^{-3}	*t* / min
0.00	0.000	0
0.0042	0.010	25
0.0061	0.025	15
0.037	0.074	30
0.85	3.4	15
2.2	4.5	30
4.4	8.8	30
5.0	10	30
11	13	50
17	14	70
20	20	60
26	52	30
35	69	30
42	56	45
67	67	60
73	73	60
77	77	60
